# Surgical Outcomes of Open, Laparoscopic, and Robotic-Assisted Approaches for Stage I Endometrial Cancer: Insights From a Real-World Study by the Indian Gynecologic-Onco Study Group

**DOI:** 10.7759/cureus.90782

**Published:** 2025-08-22

**Authors:** Vandana Jain, Jaydip Bhaumik, Rama Joshi, Yogesh Kulkarni, Kanika B Modi, Priya Bhati, Surender Dabas, Rohit R Ranade, Nidhi Nayyar, Sudhir Rawal, Harit Chaturvedi, Priya Kapoor, Arunava Roy, Swapna Misra, Somashekhar S. P., Kaustav Basu, Tarini Sonwani, Venkat P., Anik Ghosh, Basumita Chakraborti, Jagannath Mishra, Subhashree Rout, Krishna Bharadwaj, Divya Gupta, Akhil Dahiya

**Affiliations:** 1 Department of Gynecologic Oncology, Rajiv Gandhi Cancer Institute and Research Center, New Delhi, IND; 2 Department of Gynecologic Oncology, Tata Medical Center, Kolkata, IND; 3 Department of Gynecologic Oncology and Robotic Surgery, Fortis Memorial Research Institute, Gurugram, IND; 4 Department of Gynecologic Oncology and Robotic Surgery, Kokilaben Dhirubhai Ambani Hospital and Medical Research Institute, Mumbai, IND; 5 Department of Gynecologic Oncology, Max Super Specialty Hospital, Saket, New Delhi, IND; 6 Department of Gynecologic Oncology, Amrita Institute of Medical Sciences and Research Center, Kochi, IND; 7 Department of Surgical Oncology, Dr. B. L. Kapur Memorial Hospital, New Delhi, IND; 8 Department of Gynecologic Oncology and Robotic Surgery, Mazumdar Shaw Medical Center, Bengaluru, IND; 9 Department of Gynecologic Surgical Oncology and Robotic Surgery, Dr. B. L. Kapur Memorial Hospital, New Delhi, IND; 10 Department of Genito Uro Oncology Services, Rajiv Gandhi Cancer Institute and Research Center, New Delhi, IND; 11 Department of Oncology, Max Super Specialty Hospital, Saket, New Delhi, IND; 12 Department of Gynecologic Oncology, Apollo Cancer Center, Chennai, IND; 13 Department of Gynecologic Oncology and Women Cancer Initiative, Medica Superspecialty Hospital, Kolkata, IND; 14 Department of Obstetrics and Gynecology, Fortis Hospital, Mohali, IND; 15 Department of Surgical and Gynecologic Oncology and Robotic Surgery, Aster CMI Hospital, Bengaluru, IND; 16 Department of Gynecologic Oncology and Robotic Surgery, Narayana Superspeciality Hospital, Kolkata, IND; 17 Department of Surgical Oncology, Apollo Cancer Center, Chennai, IND; 18 Department of Clinical Affairs, Intuitive Surgical, Sunnyvale, USA; 19 Department of Clinical Operations, Catalyst Clinical Services Pvt. Ltd., New Delhi, IND; 20 Department of Clinical and Medical Affairs, Intuitive Surgical, Sunnyvale, USA

**Keywords:** endometrial cancer, minimally invasive surgery, perioperative outcomes, robotic-assisted surgery, staging surgery

## Abstract

Background: There is limited clinical evidence from India comparing robotic-assisted surgery (RAS), laparoscopic surgery (LPS), and open laparotomy (OL) approaches for managing stage 1 endometrial cancer (EC). This multicenter study aimed to evaluate and compare the perioperative and short-term outcomes of these techniques across high-volume Indian institutions.

Methodology: This multicenter, retrospective, non-randomized comparative study reviewed medical records of consecutive patients who underwent OL, LPS, or RAS for stage 1 EC between January 2010 and December 2023.

Results: A total of 2,090 patient records were analyzed, including 1,223 in the RAS group, 451 in the LPS group, and 416 in the OL group. The average age was 59.66 ± 9.31 years, with no significant differences between groups. The overall mean operative time was 220.51 ± 93.73 minutes, with the RAS group demonstrating a significantly shorter duration than both OL and LPS groups (*P *< 0.001). The mean estimated blood loss was 128.87 ± 226.75 mL, with the RAS group resulting in the least blood loss, followed by the LPS and OL groups (*P *< 0.001). The need for intraoperative blood transfusion was also lowest in the RAS group. Intraoperative complications were more frequent in the OL group, while conversion to open surgery was higher in the LPS group (*P *< 0.001). Hospital stay was shortest in the RAS group (2.71 days), followed by the LPS (3.77 days) and OL (5.95 days) groups (*P *< 0.001). Postoperative complications were significantly fewer in the RAS and LPS groups than in the OL group. Positive margins were lowest in the RAS group (*P *< 0.001). Sentinel lymph node sampling was highest in the RAS group (*P *< 0.001). Patients in the RAS group started adjuvant treatment sooner than the LPS (*P *< 0.001) and OL (*P *= 0.004) groups.

Conclusions: This study reports the first multicentric evidence for RAS in Indian settings, compared to conventional approaches, and offers encouraging outcomes.

## Introduction

Endometrial cancer (EC) is the sixth most common gynecologic malignancy worldwide, with its incidence steadily increasing [1]. In India, EC is the third most common gynecologic malignancy after cervical and ovarian cancers. Although its age-adjusted incidence is relatively low, it contributes notably to cancer-related mortality [2].

Doppler ultrasound, particularly when combined with transvaginal color Doppler, can assess tumor vascular flow and, through parameters such as endometrial thickness and uterine artery indices, help distinguish benign from malignant changes and predict advanced stages of EC [3-4]. Well-known risk factors such as body mass index (BMI), diabetes, and age, when combined with sonographic findings, can aid in accurately predicting EC and guiding decisions on further invasive procedures [5]. Most ECs (75%-80%) are diagnosed at FIGO (International Federation of Gynecology and Obstetrics) stage I, where key prognostic factors include stage, grade, and depth of myometrial invasion [6]. Additional factors include age, histological type, peritoneal cytology, vascular invasion, hormone receptor status, menopausal status, and uterine size. Surgery is the mainstay of treatment for early-stage EC, serving both to remove the tumor and to assess the extent of disease for planning adjuvant therapy [7]. Minimally invasive approaches like robotic-assisted surgery (RAS) or laparoscopic surgery (LPS) are preferred. Total hysterectomy with salpingo-oophorectomy (THBSO) and pelvic lymph node dissection (PLND) is the gold standard for early-stage disease [1,8]. Para-aortic nodes are removed in high-risk cases. Adjuvant radiotherapy is recommended for high-risk cases, such as those with deep myometrial invasion or high-grade tumors, to reduce the risk of recurrence [6].

Traditionally, early-stage EC was treated with open surgery (THBSO with or without lymphadenectomy), but this approach often led to complications, particularly in obese or diabetic patients [9]. LPS, introduced in the 1990s, offered a less invasive alternative with reduced blood loss, fewer complications, and faster recovery, though its adoption has been limited by technical challenges [10-12]. The introduction of RAS in 2005 improved precision and ease, especially benefiting high-risk groups like obese patients [9,13]. While global data support RAS and LPS over open laparotomy (OL), evidence from India remains limited. The objective of this study was to compare operative outcomes, including operative time, estimated blood loss, hospital stay, complications, conversions, positive surgical margins, and lymph node yield, across different surgical approaches for stage I EC in multiple high-volume Indian centers.

## Materials and methods

This retrospective study was conducted across 13 Indian hospitals, reviewing records of women over 18 who underwent OL, LPS, or RAS for pathologically confirmed stage 1 EC between January 2010 and December 2023. The staging system used was the 2017 American Joint Committee on Cancer (AJCC) 8th Edition Tumor-Node-Metastasis (TNM) classification and the FIGO surgical staging system for EC. Eligible procedures included THBSO, peritoneal lavage, lymph node dissection, adhesiolysis, or sentinel node mapping. The study aimed to assess perioperative outcomes until discharge and short-term clinical outcomes up to 90 days post-surgery. The study adhered to the latest Helsinki Declaration and Good Clinical Practice guidelines, with ethics approval from all participating centers. It was registered with the Clinical Trials Registry of India (CTRI/2023/11/060042).

De-identified data were extracted from hospital records, operative notes, anesthesia charts, and pathology reports and entered into a structured case record proforma that was managed using Microsoft Excel. Variables collected included preoperative factors (age, BMI, parity, comorbidities, and history of previous surgeries), intraoperative details (surgical approach, operative time, blood loss, transfusion requirements, intraoperative complications, and conversions), postoperative outcomes (length of stay, and complications), and histopathological findings (tumor type, lymph node yield, sentinel node sampling, and margin status). Post-discharge information on adjuvant therapy, recurrence, where available, and mortality within 90 days was also recorded. To maintain uniformity, each center appointed a principal investigator and trained research staff, with data collection standardized through unified training. The study was approved by the institutional ethics committees of all participating centers, and a waiver of informed consent was granted, given the retrospective nature of de-identified data collection.

Statistical analysis

Categorical variables were summarized as frequencies and percentages, while quantitative data were assessed for approximate normality and presented as mean (standard deviation (SD)) or median (min, max) accordingly. Categorical variables were compared using the Chi-square test or Fisher’s exact test, as appropriate. Means across the three groups were analyzed using one-way analysis of variance (ANOVA), with post hoc tests conducted when needed. The Kruskal-Wallis test was used to compare medians between groups. If a significant overall difference was found, pairwise comparisons were performed using Bonferroni-adjusted *P*-values. Analysis was performed using Stata v16.0 (StataCorp LLC, College Station, TX), with significance set at *P* < 0.05 (or *P* < 0.016 for Bonferroni adjustments).

## Results

Descriptive characteristics of preoperative variables

**Figure 1:**
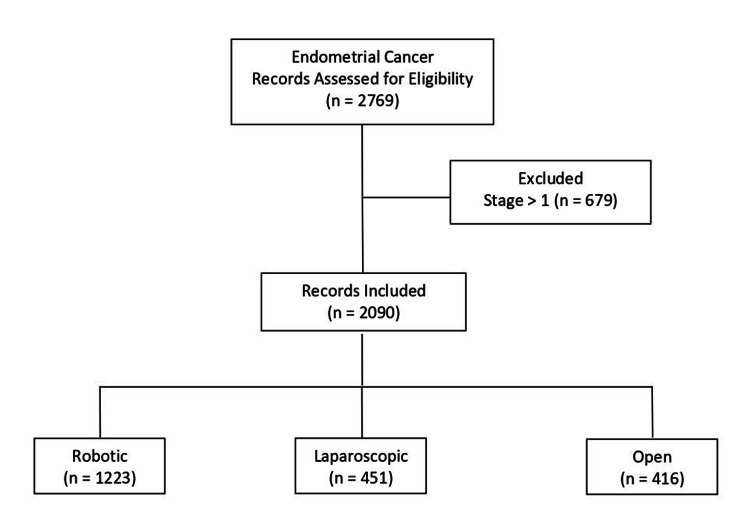
Study flowchart.

Data from 2,090 patients were analyzed: 1,223 underwent RAS, 451 LPS, and 416 OL (Figure 1).

The average age was 59.66 ± 9.31 years, with no significant age differences across groups. The RAS group had a significantly higher mean BMI than the LPS and OL groups. Parity was also higher in the RAS and OL groups than in the LPS group. Comorbidities were similar, except for chronic kidney disease, which was more common in the LPS group than in the RAS group (*P *= 0.008). A significant difference in prior abdominal surgeries was noted between the RAS and LPS groups (*P* = 0.034). Differences in previous gynecological surgeries were also significant between RAS and LPS (*P *< 0.001), and between LPS and OL groups (*P* < 0.001) as determined by the Pearson’s chi-square test. Additionally, prior obstetric surgery was more common in the RAS (*P* = 0.002) and LPS (*P* = 0.005) groups compared to the OL group using the same test. Preoperative characteristics are detailed in Table 1.

**Table 1:** Descriptive characteristics of preoperative variables. *Significant value. Statistical methods: Chi-square test, Fisher’s exact test, analysis of variance (ANOVA), Kruskal-Wallis test, and Bonferroni RAS, robotic-assisted surgery; LPS, laparoscopic surgery; OL, open laparotomy; SD, standard deviation; BMI, body mass index

Variable	Overall (*N* = 2,090)	RAS (*N* = 1,223)	LPS (*N* = 451)	OL (*N* = 416)	*P*-value		
					RAS vs. LPS	RAS vs. OL	LPS vs. OL
Age, mean ± SD, year	59.66 ± 9.31	59.75 ± 9.55	60.07 ± 8.85	58.98 ± 9.03	1.000	0.437	0.251
BMI, mean ± SD, kg/m^2^	30.68 ± 6.04	31.43 ± 6.40	29.68 ± 5.28	30.03 ± 5.70	<0.001*	0.004*	1.000
Parity, mean ± SD	2.24 ± 1.29	2.29 ± 1.30	1.99 ± 1.17	2.29 ± 1.33	0.006*	1.000	0.027
Parity, median (range)	2 (0-9)	2 (0-9)	2 (0-7)	2 (0-8)	0.006*	1.000	0.027
Co-morbidities, *n* (%)							
Hypertension	1,155 (55.3)	672 (54.9)	255 (56.5)	228 (54.8)	0.560	0.961	0.608
Diabetes	708 (33.9)	416 (34.0)	158 (35.0)	134 (32.2)	0.697	0.501	0.380
Chronic kidney disease	27 (1.3)	10 (0.8)	11 (2.4)	6 (1.4)	0.008*	0.263	0.290
Chronic liver disease	13 (0.6)	9 (0.7)	3 (0.7)	1 (0.2)	0.879	0.262	0.356
Previous abdominal surgery, *n* (%)	132 (6.3)	66 (5.4)	37 (8.2)	29 (7.0)	0.034*	0.235	0.494
Previous gynecological surgery, *n* (%)	250 (12.0)	112 (9.2)	101 (22.4)	37 (8.9)	<0.001*	0.872	<0.001*
Previous obstetric surgery, *n* (%)	285 (13.6)	180 (14.7)	68 (15.1)	37 (8.9)	0.854	0.002*	0.005*

Intraoperative outcomes of the study population

Table 2 summarizes the intraoperative outcomes.

**Table 2:** Intraoperative outcomes of the study population. *Significant value. Statistical methods: Chi-square test, Fisher’s exact test, analysis of variance (ANOVA), Kruskal-Wallis test, and Bonferroni RAS, robotic-assisted surgery; LPS, laparoscopic surgery; OL, open laparotomy; SD, standard deviation

Variable	Overall (*N *= 2,090)	RAS (*N *= 1,223)	LPS (*N *= 451)	OL (*N *= 416)	*P*-value		
					RAS vs. LPS	RAS vs. OL	LPS vs. OL
Operative time, mean ± SD, minutes	220.51 ± 93.73	205.65 ± 87.60	257.06 ± 109.41	224.66 ± 79.22	<0.001*	0.005*	<0.001*
Estimated blood loss, mean ± SD, mL	128.87 ± 226.75	54.55 ± 84.63	111.42 ± 150.48	360.31 ± 383.86	<0.001*	<0.001*	<0.001*
Estimated blood loss, median (IQR), mL	50 (5-150)	20 (5-100)	50 (Nil-200)	300 (150-450)	<0.001*	<0.001*	<0.001*
Intraoperative blood transfusion, *n* (%)	71 (3.4)	13 (1.1)	11 (2.4)	47 (11.3)	0.036*	<0.001*	<0.001*
Intraoperative complications, *n* (%)	21 (1.0)	7 (0.6)	7 (1.6)	7 (1.7)	0.051	0.034*	0.879
Conversion to open, *n* (%)	10 (0.5)	2 (0.2)	8 (1.8)	-	<0.001*	-	-

The overall mean operative time was 220.51 ± 93.73 minutes, with RAS showing the shortest duration compared to both OL and LPS (*P *< 0.001). LPS had a significantly longer operative time than OL (*P* = 0.005). Mean estimated blood loss was 128.87 ± 226.75 mL, with RAS associated with the least blood loss, followed by LPS and OL (all comparisons *P* < 0.001). Intraoperative blood transfusions were required in 3.4% of cases, lowest in the RAS group (1.1%), followed by LPS (2.4%) and OL (11.3%), with statistically significant differences across all groups. Intraoperative complications occurred in 1.0% of patients, significantly more in the OL group than in RAS (*P* = 0.034). Additionally, conversion to open surgery was more frequent in the LPS group compared to RAS (*P* < 0.001).

Postoperative outcomes and histopathological findings of the study population

Postoperative outcomes and histopathological findings are summarized in Table 3.

**Table 3:** Postoperative outcomes of the study population. *Significant value. Statistical methods: Chi-square test, Fisher’s exact test, analysis of variance (ANOVA), Kruskal-Wallis test, and Bonferroni RAS, robotic-assisted surgery; LPS, laparoscopic surgery; OL, open laparotomy; SD, standard deviation

Variable	Overall (*N *= 2,090)	RAS (*N *= 1,223)	LPS (*N *= 451)	OL (*N *= 416)	*P*-value		
					RAS vs. LPS	RAS vs. OL	LPS vs. OL
Length of hospital stay, mean ± SD, days	3.58 ± 3.05	2.71 ± 1.81	3.77 ± 4.48	5.95 ± 2.76	<0.001*	<0.001*	<0.001*
Postoperative complications, *n* (%)	43 (2.1)	16 (1.3)	7 (1.6)	20 (4.8)	0.704	<0.001*	0.006*
Clavien-Dindo classification, *n* (%)							
Grade I	21 (48.8)	4 (25.0)	5 (71.4)	12 (60.0)	0.066	0.049*	0.590
Grade II	12 (27.9)	6 (37.5)	1 (14.3)	5 (25.0)	0.366	0.418	0.498
Grade III	8 (18.6)	5 (31.3)	1 (14.3)	2 (10.0)	0.621	0.204	0.610
Grade IV	2 (4.7)	1 (6.3)	0	1 (5.0)	0.696	0.698	0.741
Histological type, *n* (%)							
Endometroid	1871 (89.5)	1105 (90.4)	412 (91.4)	354 (85.1)	0.533	0.003*	0.004*
Serous carcinoma	111 (5.3)	60 (4.9)	22 (4.9)	29 (7.0)	0.981	0.108	0.191
Clear cell carcinoma	28 (1.3)	13 (1.1)	5 (1.1)	10 (2.4)	0.936	0.045*	0.144
Carcinosarcoma	56 (2.7)	34 (2.8)	10 (2.2)	12 (2.9)	0.523	0.911	0.532
Mesenchymal tumor type	12 (0.6)	3 (0.2)	1 (0.2)	8 (1.9)	0.930	0.000*	0.014*
Not available or not documented	12 (0.6)	8 (0.7)	1 (0.2)	3 (0.7)	0.283	0.885	0.278
Positive surgical margin, *n* (%)	7 (0.3)	1 (0.1)	5 (1.1)	1 (0.2)	<0.001*	0.694	<0.001*
Total number of lymph nodes harvested, mean ± SD	18.10 ± 11.45	17.27 ± 10.90	16.65 ± 11.99	22.09 ± 11.55	1.000	<0.001*	<0.001*
Sentinel lymph nodes sampling, *n* (%)	655 (31.3)	487 (39.8)	146 (32.4)	22 (5.3)	0.005*	<0.001*	<0.001*

The RAS group had the shortest hospital stay (2.71 ± 1.81 days), significantly less than both LPS (3.77 ± 4.48 days; *P* < 0.001) and OL (5.95 ± 2.76 days; *P* < 0.001). LPS patients also had shorter stays than those undergoing OL (*P* < 0.001). Postoperative complications occurred in 1.3% (RAS), 1.6% (LPS), and 4.8% (OL) of patients, with significantly fewer events in RAS vs. OL (*P* < 0.001) and LPS vs. OL (*P* = 0.006). The RAS group experienced complications such as abdominal distension, constipation, fever, left ureteric leak, urinary retention, vaginal bleeding, and vertigo. In comparison, the LPS group had fever, ileus, mild incisional pain, and postoperative abnormal heart rhythm, while the OL group reported abdominal pain, bradycardia, breathlessness, constipation, fever, ileus, intra-abdominal hemorrhage, mild distension, urinary retention, and vomiting. All patients had confirmed malignancy, predominantly endometrioid carcinoma, followed by serous, carcinosarcoma, clear cell, and mesenchymal types. Positive surgical margins were rare (0.3%) and lowest in the RAS group, significantly less than in LPS (*P* < 0.001). The OL group also showed fewer positive margins than LPS (*P* < 0.001). Sentinel lymph node sampling was highest in the RAS group compared to both LPS (*P* = 0.005) and OL (*P* < 0.001), with LPS also outperforming OL in this regard (*P* < 0.001).

Short-term follow-up outcomes of the study population

Short-term follow-up outcomes are presented in Table 4.

**Table 4:** Short-term follow-up outcomes of the study population (from surgery up to 90 days). *Significant value. Statistical methods: Chi-square test, Fisher’s exact test, analysis of variance (ANOVA), Kruskal-Wallis test, and Bonferroni RAS, robotic-assisted surgery; LPS, laparoscopic surgery; OL, open laparotomy; SD, standard deviation

Variable	Overall (*N *= 1,576)	RAS (*N *= 931)	LPS (*N *= 326)	OL (*N *= 319)	*P*-value		
					RAS vs. LPS	RAS vs. OL	LPS vs. OL
Postoperative adjuvant therapy, *n* (%)	593 (37.6)	334 (35.9)	128 (39.3)	131 (41.1)	0.275	0.098	0.641
Type of adjuvant therapy, *n* (%)							
Radiation	401 (67.6)	235 (70.4)	92 (71.9)	74 (56.5)	0.749	0.004*	0.010*
Chemotherapy	110 (18.5)	52 (15.6)	21 (16.4)	37 (28.2)	0.825	0.002*	0.022*
Chemotherapy + radiation	81 (13.7)	46 (13.8)	15 (11.7)	20 (15.3)	0.560	0.678	0.404
Not available	1 (0.2)	1 (0.3)	0	0	0.723	0.718	-
Time to start of adjuvant therapy, mean ± SD, days	33.09 ± 13.14	30.31 ± 11.75	37.74 ± 14.38	34.78 ± 13.21	<0.001*	0.004*	0.216
Number of deaths, at day 90 post-surgery, *n* (%)	4 (0.3)	1 (0.1)	2 (0.6)	1 (0.3)	0.167	0.445	0.508

Adjuvant therapy was given to 37.6% of patients, with no significant overall difference between surgical groups. However, radiation therapy was more commonly administered in the RAS and LPS groups compared to OL, while chemotherapy use was significantly higher in the OL group. The RAS group also began adjuvant treatment earlier than both the LPS (*P* < 0.001) and OL (*P* = 0.004) groups. Four deaths occurred during follow-up, with no significant variation across groups.

## Discussion

EC rates have surged over the past 30 years, largely due to aging and rising obesity [14]. With earlier detection, surgery has become central to the management of EC. Minimally invasive techniques, especially RAS, are widely adopted, used in nearly 90% of cases at high-volume centers, due to better precision and outcomes [15-16]. While RAS is increasingly preferred over LPS and OL techniques, data comparing these approaches in India remains limited. This large real-world study evaluates and compares surgical and oncologic outcomes of RAS, LPS, and OL in stage 1 EC across high-volume Indian centers.

Baseline characteristics in our study mirrored global trends [17-19]. The average age in our study was 59.66 ± 9.31 years, with no significant differences across surgical groups, consistent with prior reports showing that EC commonly affects postmenopausal women, with a median diagnosis age of around 61 years [20]. Age plays a key role in disease incidence, prognosis, and treatment response, and RAS may offer added advantages such as improved accessibility and enhanced surgical precision, particularly in narrow spaces in older or high-risk patients [20]. The mean BMI was 30.68 ± 6.04 kg/m², with significantly higher values observed in the RAS group compared to LPS (*P *< 0.001) and OL (*P *= 0.004). This finding aligns with recent Indian data supporting the safety and effectiveness of RAS in both non-obese and obese patients, highlighting its utility in managing EC in those with elevated BMI [21].

Intraoperative and postoperative outcomes are critical measures of surgical safety. In our study, the RAS group had the shortest mean operative time (205.65 ± 87.60 minutes) compared to LPS (257.06 ± 109.41 minutes) and OL (224.66 ± 79.22 minutes), with both differences being statistically significant. These findings are consistent with a prior randomized trial that reported shorter operative times with RAS versus LPS (mean difference: -31 minutes, 95% confidence interval (CI) -43.23 to -18.77, *P *< 0.00001) [18]. A cohort study has also shown shorter durations for RAS (155.6 ± 45.7 minutes) and LPS (178.6 ± 58.7 minutes) when compared to OL (195.3 ± 67.0 minutes) [22]. Longer operative times in early RAS and LPS cases are often attributed to the learning curve. However, with increased experience and standardized protocols, durations typically decrease, potentially making RAS more efficient than OL [9]. Notably, in our cohort, LPS was associated with significantly longer operative times compared to OL (*P *= 0.005), consistent with findings from a systematic review reporting similar trends [1]. The review reported mean increases of 18.95 minutes (95% CI: 7.68-30.20) for LPS and 29.00 minutes (95% CI: 13.66-44.23) for RAS compared to OL, findings that differ from ours. We believe the involvement of high-volume centers in our study may have contributed to the shorter operative times observed with RAS.

In our study, the RAS group had significantly lower blood loss (54.55 ± 84.63 mL) compared to LPS (111.42 ± 150.48 mL, *P* < 0.001) and OL (360.31 ± 383.86 mL, *P *< 0.001). LPS also showed significantly less blood loss than OL (*P *< 0.001). These results align with several meta-analyses reporting reduced blood loss with RAS and LPS over OL [1,23]. Some studies also found RAS superior to LPS in minimizing blood loss [24], though others reported no significant difference [1]. The technical advantages of RAS, such as 3D-magnified vision and improved instrument control, likely contribute to lower vascular injury and bleeding [9]. Correspondingly, intraoperative transfusion rates were lowest in the RAS group, significantly lower than both LPS and OL. LPS also required fewer transfusions than OL. These findings are supported by previous meta-analyses, though some studies found no significant difference between RAS and LPS in transfusion needs [23-24]. The lower transfusion rates observed with RAS in our cohort are likely attributable to its consistently lower blood loss.

Our study observed significantly fewer intraoperative complications in the RAS group compared to OL, but no notable difference between RAS and LPS, findings that align with previous research. One study reported similar complication rates across all three groups (2.8% RAS, 8.3% LPS, 5.2% OL; *P *= 0.313) [25], and a meta-analysis also found no significant difference between RAS and LPS (RR = 0.81; 95% CI: 0.61-1.06; *P *> 0.05) [26]. In terms of conversions to open surgery, our results are consistent with published evidence. Meta-analyses have shown significantly lower conversion rates with RAS compared to LPS (odds ratio (OR) = 0.38; 95% CI: 0.21-0.67; *P *= 0.0008) [23] and (risk ratio (RR) = 0.41; 95% CI: 0.29-0.59; *P *< 0.00001) [23]. These findings underscore a key advantage of RAS in preserving minimally invasive access, particularly in complex surgical scenarios.

Our study found that the RAS group had a significantly shorter hospital stay (2.71 ± 1.81 days) compared to LPS (3.77 ± 4.48 days, *P *< 0.001) and OL (5.95 ± 2.76 days, *P *< 0.001). These results align with meta-analyses showing shorter stays with RAS over LPS and OL [23-26], and a systematic review reporting a mean reduction of 3.79 days with RAS and 3.54 days with LPS compared to OL [1]. The faster recovery with RAS is likely due to reduced surgical trauma, less postoperative pain, and earlier return to normal activity [26]. Postoperative complications were also lower in the RAS group, consistent with prior studies showing reduced complication rates with both RAS (OR = 0.46) and LPS (OR = 0.48) versus OL [1]. Another study found LPS had significantly fewer complications than OL, with similar rates between RAS and LPS [25]. While some meta-analyses reported slightly lower complication and transfusion rates with RAS compared to LPS, differences were not statistically significant [27].

Comprehensive lymphadenectomy plays a vital role in EC staging, with improved overall survival linked to a higher number of lymph nodes removed, highlighting its prognostic value and its use as a marker of surgical quality [28-29]. In our study, sentinel lymph node sampling was performed most frequently in the RAS group, significantly more than in the LPS (*P *= 0.005) and OL (*P *< 0.001) groups. LPS also showed a higher rate than OL (*P *< 0.001), highlighting the technical benefits of minimally invasive surgical approaches. While the overall rate of postoperative adjuvant therapy was similar across groups, the RAS group initiated treatment significantly earlier than LPS (*P *< 0.001) and OL (*P *= 0.004), suggesting a faster recovery and surgical benefit with RAS. This is particularly important, as studies have shown that delays in starting adjuvant radiation beyond eight weeks after surgery may increase the risk of recurrence in early-stage EC [30]. Although adjuvant therapy in all three groups of our study began within five weeks, long-term oncologic outcomes remain to be evaluated.

Limitations

This study has several limitations. Variability in surgeon participation across centers may have influenced outcomes, though it enhances the external validity compared to single-center analyses. Differences in surgical experience and learning curves, particularly for RAS and LPS, were not captured and may have affected perioperative results. As a retrospective study, it is inherently subject to selection bias, as the choice of surgical approach was not randomized but likely influenced by patient characteristics (e.g., age, BMI, comorbidities), tumor features (e.g., size, histology), and institutional expertise. These factors could have predisposed certain patients to receive one approach over another, thereby impacting observed outcomes. Moreover, unmeasured confounders such as socioeconomic status, surgeon preference, perioperative care practices, and center-level resource availability were not accounted for, which may have introduced residual bias despite standardized data collection. The lack of prospective follow-up further precluded the assessment of long-term oncological outcomes, limiting the ability to evaluate recurrence and survival. Despite these constraints, the study provides valuable real-world insights into surgical outcomes for stage 1 EC across high-volume Indian centers. While a prospective randomized trial would provide stronger evidence, this study adds meaningful data on the practical use and growing role of RAS for EC within the Indian healthcare setting.

## Conclusions

This multicenter, retrospective, real-world study suggests a potential role for RAS in the management of stage I EC. The RAS arm was associated with trends toward shorter operative time, reduced blood loss, fewer complications, faster recovery, and earlier initiation of adjuvant therapy compared with LPS and OL. Margin positivity also appeared lower in the RAS arm than in LPS. These findings support the possible clinical advantages of minimally invasive approaches, particularly RAS, in early-stage EC, though causality cannot be established given the retrospective design. The results may provide useful insights to guide Indian surgeons in routine clinical practice; however, further prospective, long-term studies are warranted to more definitively assess the oncological outcomes and broader benefits of adopting advanced RAS techniques.
